# Effect of Austempering on the Microstructure, Mechanical Properties and Tribological Performance of EN-GJS-800-8 Ductile Iron Castings

**DOI:** 10.3390/ma19163472

**Published:** 2026-08-17

**Authors:** Jarosław Jaroszek, Edward Miko, Łukasz Nowakowski, Artur Zaczyński

**Affiliations:** 1 Faculty of Mechatronics and Machine Design, Kielce University of Technology, Tysiąclecia Państwa Polskiego 7, 25-314 Kielce, Poland; emiko@tu.kielce.pl (E.M.); lukasn@tu.kielce.pl (Ł.N.); 2 Odlewnie Polskie S.A., Inżyniera Władysława Rogowskiego 22, 27-200 Starachowice, Poland; artur.zaczynski@odlewniepolskie.pl

**Keywords:** austempered ductile iron (ADI), EN-GJS-800-8, austempering, ausferritic matrix, quantitative metallography, mechanical properties, wear resistance, tribology

## Abstract

This study evaluated the effect of austempering on the microstructure, mechanical properties, and dry-sliding wear performance of EN-GJS-800-8 ductile iron produced as cross-step castings with wall thicknesses of 8, 17, 38, and 48 mm. As-cast and heat-treated specimens were examined by optical microscopy and quantitative image analysis, Brinell hardness, tensile and Charpy impact testing, ball-on-disk testing, and three-dimensional confocal profilometry. Austenitization at 880–890 °C for 2.5 h followed by austempering at 370–380 °C for 2 h produced an optically homogeneous acicular matrix consistent with ausferrite in all investigated sections and reduced the wall-thickness-related variation in hardness. The average hardness reached 296.4 HBW, the ultimate tensile strength 935 MPa, and elongation 8.6%. The mean Charpy impact energy increased from 5.12 to 10.06 J. The wear-track cross-sectional area decreased from approximately 2.98 × 10^4^ to 210 μm^2^, while the maximum track depth decreased from 43.5 to 3 μm. The selected heat-treatment cycle therefore improved strength, impact-energy absorption, and wear resistance while promoting a more uniform response across the casting sections.

## 1. Introduction

Ductile iron is widely used for engineering castings because it combines low production cost, excellent castability, good machinability, and attractive mechanical properties. Spheroidal graphite reduces stress concentration relative to gray cast iron, thereby improving tensile strength, ductility, fatigue resistance, and fracture toughness. Consequently, ductile irons and ADI are used in demanding engineering applications [1,2]. The austempering-induced microstructure strongly influences their mechanical and tribological performance [3,4].

Among the commercially available grades, EN-GJS-800-8 offers a favorable balance between strength and ductility. According to EN 1563 [5], the grade combines a minimum tensile strength of 800 MPa with a minimum elongation of 8%. Wall thickness and mould conditions can additionally affect the structure and mechanical properties of ductile iron castings [6].

Austempering can further improve the performance of ferritic–pearlitic ductile iron. Austenitization followed by isothermal transformation produces an ausferritic matrix, commonly described as acicular ferrite and carbon-enriched retained austenite. Depending on the heat-treatment parameters, this microstructure can provide a favorable combination of strength, ductility, fatigue resistance, toughness, and wear resistance [1,7,8,9,10].

The final response of ADI depends on both the austempering cycle and the initial casting microstructure. Wall thickness controls the local solidification rate and therefore affects graphite nodule density, ferrite-to-pearlite ratio, segregation, and hardness before heat treatment. These local differences may alter transformation kinetics and produce property gradients in complex castings if the thermal cycle is not appropriately selected [6,8,11,12,13].

Recent studies have also shown that the tribological response of ADI cannot be explained by hardness alone; strain hardening, graphite transfer, contact conditions, and the stability of retained austenite may contribute to wear behavior [9,14,15,16,17]. However, conclusions concerning retained-austenite transformations require direct phase analysis, such as X-ray diffraction or electron backscatter diffraction, rather than optical microscopy alone [10,15,18].

Despite extensive research on ADI, relatively few studies have integrated quantitative metallography, hardness, tensile behavior, impact energy, and three-dimensional wear-track characterization for EN-GJS-800-8 cross-step castings with different wall thicknesses. The novelty of the present work is the combined assessment of casting geometry, graphite morphology, matrix evolution, mechanical performance, and dry-sliding wear under an identical chemical composition and processing history.

The objective of this study was therefore to determine whether the selected austempering cycle could reduce microstructural and property differences caused by wall-thickness-dependent cooling conditions. Particular attention was given to quantitative microstructural parameters, hardness uniformity, tensile properties, Charpy impact energy, and wear-track geometry.

## 2. Materials and Methods

### 2.1. Material Characterization and Chemical Composition

**Figure 1:**
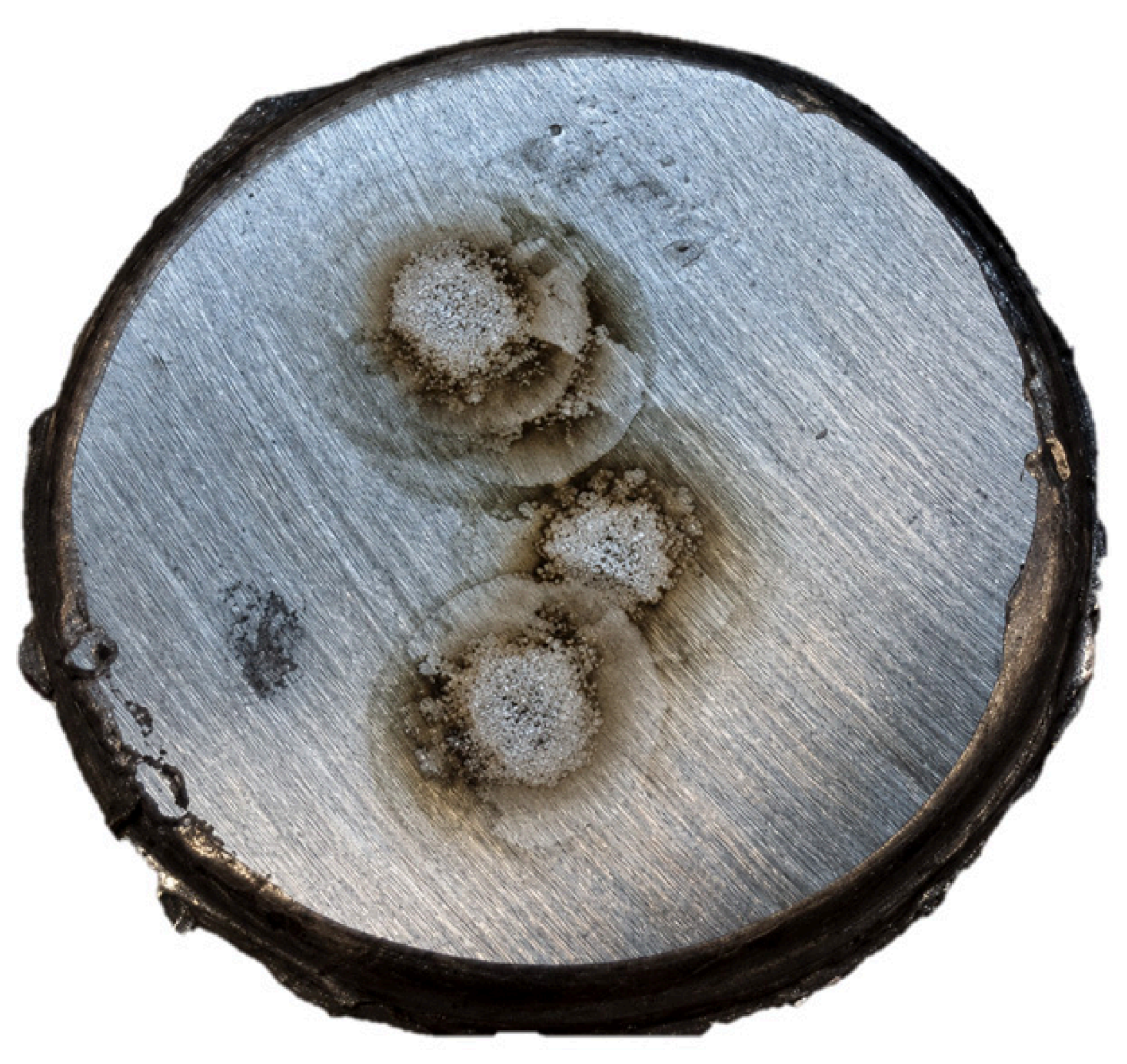
Representative specimen used for optical emission spectroscopy (OES), showing the spark burn marks. The specimen diameter was 40 mm and its thickness was 3.9 mm.

The investigated material was ductile iron produced under industrial foundry conditions and designed to obtain the EN-GJS-800-8 ADI grade after austempering heat treatment. To verify the chemical composition of the alloy and its suitability for the intended ADI grade, optical emission spectroscopy (OES) was performed using a Spectrolab M10 spark emission spectrometer (SPECTRO, Kleve, Germany). A representative specimen used for chemical analysis is shown in Figure 1.

The average chemical composition obtained from four independent melts is presented in Table 1.

### 2.2. Experimental Castings and Sample Preparation

Experimental castings were produced in standardized sand molds using a dedicated cross-step casting geometry designed to generate sections with different cooling rates. This configuration enables the assessment of structural homogeneity and casting quality across regions subjected to varying solidification conditions.

The casting design incorporated four wall thicknesses: 8, 17, 38, and 48 mm. The use of this geometry allows simultaneous evaluation of the influence of cooling rate on graphite nodularity, matrix formation in the as-cast condition, and the subsequent response of the material to austempering treatment.

**Figure 2:**
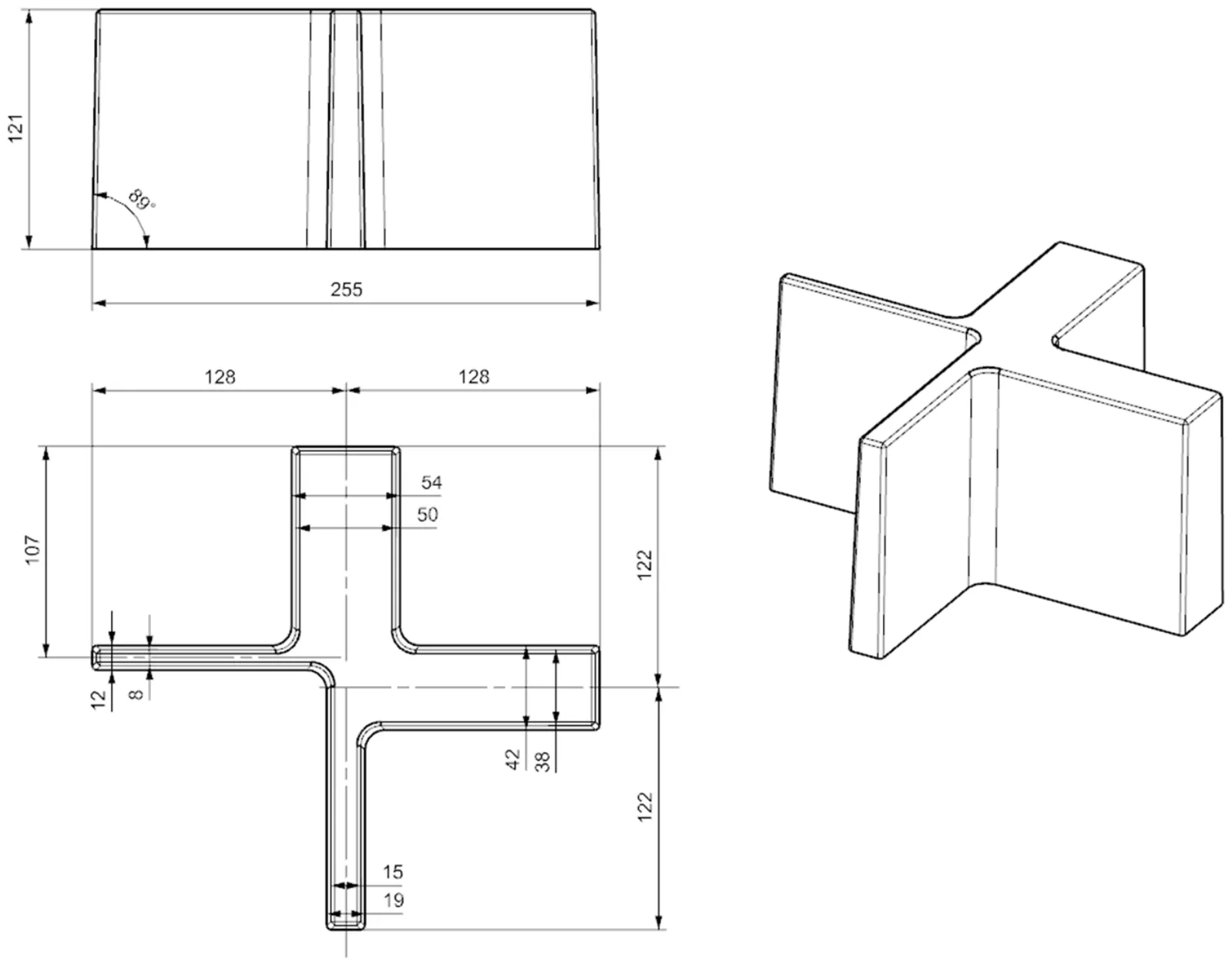
Schematic representation of the cross-step casting showing wall thicknesses of 8, 17, 38, and 48 mm.

Specimens for metallographic characterization and mechanical testing were extracted from the central region of each wall section to minimize edge effects and ensure representative sampling. This approach enabled a systematic investigation of the influence of section thickness on casting quality prior to subsequent machining studies. A test casting with different wall thicknesses is shown in Figure 2.

### 2.3. Austempering Heat Treatment

To transform the conventional ferritic–pearlitic matrix into a high-strength ausferritic structure, the castings were subjected to a two-stage austempering heat treatment. Thermal cycles were performed in an industrial chamber furnace equipped with programmable microprocessor controllers ensuring temperature stability within ±2 °C.

The heat-treatment cycle consisted of the following stages:

1. Austenitization: Samples were heated to 880–890 °C and held for 2.5 h to achieve complete austenitization and homogeneous carbon distribution.
2. Isothermal Quenching (Austempering): Immediately after austenitization, the specimens were transferred to a molten salt bath maintained at 370–380 °C and held for 2.0 h. This stage promoted the formation of acicular ferrite and carbon-enriched retained austenite.
3. Final Cooling: After austempering, the castings were removed from the salt bath and cooled to room temperature in still air.

### 2.4. Metallographic Examination and Quantitative Analysis

#### 2.4.1. Specimen Preparation and Optical Microscopy

Metallographic investigations were performed to evaluate the microstructural evolution before and after austempering.

Samples were cut from the individual wall-thickness sections, ground with silicon carbide abrasive papers to 2000 grit, and polished using a 1 μm diamond suspension. Graphite morphology was evaluated on polished, unetched specimens. Matrix morphology was examined after etching with 3% Nital (3 vol.% HNO_3_ in ethanol). Observations were performed using a Nikon Eclipse MA100 inverted optical microscope (Nikon Corporation, Tokyo, Japan) equipped with a DFKNME33UX264 digital camera (The Imaging Source Asia, Taipei, Chinese) and NIS-Elements Basic Research software (version 5.21.03).

Representative fields were obtained from the central regions of the 8, 17, 38, and 48 mm sections in both material conditions. The unetched images were used for graphite characterization, whereas the etched images were used to assess matrix constitution and microstructural homogeneity.

#### 2.4.2. Quantitative Metallographic Analysis

Quantitative image analysis was performed on calibrated optical micrographs using NIS-Elements Basic Research. Graphite nodule density, graphite area fraction, and nodularity were determined from polished, unetched specimens. Ferrite and pearlite fractions were determined from 3% Nital-etched images of the as-cast material. In the austempered condition, optical microscopy was used to evaluate the morphology and uniformity of the transformed matrix; retained-austenite content was not quantified.

(1) $$N_{A}=\frac{N}{A}$$

(2) $$G=\left(\frac{A_{G}}{A}\right)\times 100\%$$

(3) $$N_{od}=\left(\frac{N_{sph}}{N_{tot}}\right)\times 100\%$$

(4) $$F=\left(\frac{A_{F}}{A_{M}}\right)\times 100\%$$

(5) $$P=\left(\frac{A_{P}}{A_{M}}\right)\times 100\%$$

where *N*_A_ is the graphite nodule density (mm^−2^), *N* is the number of graphite nodules, *A* is the analyzed image area, A_G_ is the graphite area, *N*_sph_ is the number of spheroidal graphite particles, *N*_tot_ is the total number of graphite particles, *A*_F_ and *A*_P_ are the ferrite and pearlite areas, respectively, and *A*_M_ is the analyzed matrix area excluding graphite.

The arithmetic mean, sample standard deviation, 95% confidence interval, and percentage reduction in wear-track cross-sectional area were calculated as follows:

(6) $$\bar{x}\;=\;\frac{1}{n}\;\sum_{i\;=\;1}^{n}x_{i}$$

(7) $$SD=\sqrt{\frac{\sum_{i=1}^{n}{\left(x_{i}-\bar{x}\right)}^{2}}{\left(n-1\right)}}$$

(8) $${CI}_{95\%}=\bar{x}\;\pm \;t_{0.975,n-1}\;\cdot \;\frac{SD}{\sqrt{n}}$$

(9) $$WR=\left[\frac{A_{w,AC}-A_{w,ADI}}{A_{w,AC}}\right]\times 100\%$$

In Equations (6)–(9), n is the number of measurements, t is Student’s t coefficient, and *A_w_*_,AC_ and *A_w_*_,ADI_ are the wear-track cross-sectional areas in the as-cast and austempered conditions. Five hardness measurements were used for each wall thickness. Because the tensile and Charpy tests comprised two specimens per material condition, those results are reported descriptively and no inferential significance test is claimed.

### 2.5. Mechanical Testing Procedures

Mechanical testing was performed at room temperature (18–22 °C) to correlate microstructural changes with mechanical performance.

#### 2.5.1. Brinell Hardness Testing

Brinell hardness measurements were carried out using an Innovatest Nexus 3300 hardness tester (Innovatest, Maastricht, The Netherlands). A 10 mm tungsten carbide ball indenter was applied under a test force of 29.42 kN (3000 kgf) with a dwell time of 15 s, in accordance with ISO 6506-1 [19]. For each material condition and wall-thickness section, five independent indentations were performed. The results are presented as the mean value, standard deviation (SD), and 95% confidence interval (95% CI), with the latter calculated using Student’s *t*-distribution.

Hardness measurements were performed on specimens extracted from all four wall-thickness sections (8, 17, 38, and 48 mm).

#### 2.5.2. Tensile Testing

Uniaxial tensile tests were conducted on specimens in both the as-cast and austempered conditions using a Zwick/Roell Z250 (ZwickRoell Group, Ulm, Germany) universal testing machine equipped with a 250 kN load cell.

Testing procedures followed ISO 6892-1 [20]. Tensile tests were carried out on two proportional cylindrical specimens for each material condition according to ISO 6892-1. The specimens had a gauge diameter of 10 mm and a gauge length of 50 mm. The tests were performed under displacement control with a crosshead speed of 1 mm/min, corresponding to the strain rate specified in the standard. Due to the geometry of the step casting and the dimensional requirements specified in ISO 6892-1, the tensile specimens were machined so that the gauge length extended across the 17 mm and 48 mm wall-thickness sections.

#### 2.5.3. Charpy Impact Testing

Charpy V-notch impact tests were performed using a HOYTOM HYTT 300 J (Hoytom S.L., Leioa, Spain) pendulum impact tester with a maximum impact energy capacity of 300 J.

Two standard Charpy V-notch specimens with dimensions of 10 × 10 × 55 mm were tested for each material condition according to ISO 148-1 [21]. The notch orientation was perpendicular to the casting surface, and the tests were conducted at room temperature (approximately 20 °C). The absorbed Charpy impact energy was recorded and used as an indicator of impact resistance and energy absorption capability.

**Figure 3:**
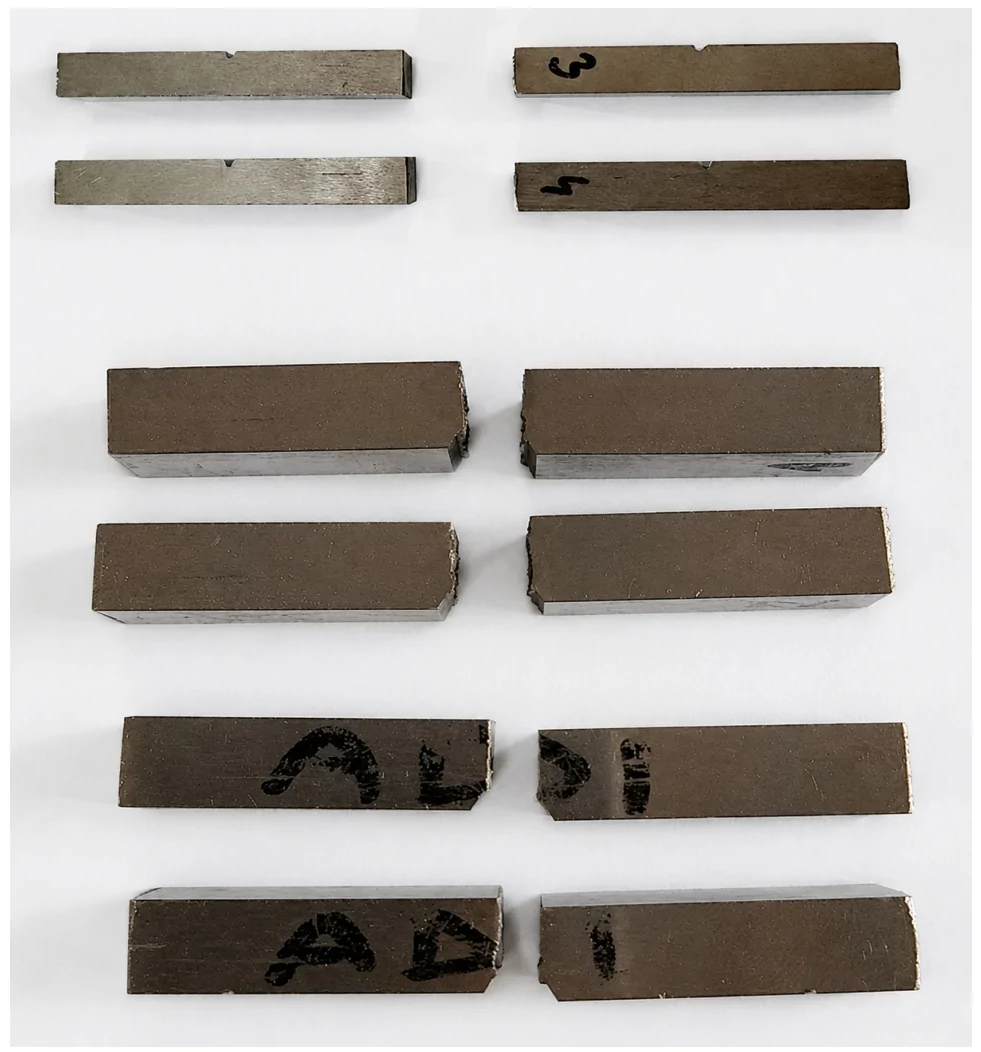
Standard Charpy V-notch specimens (10 × 10 × 55 mm) before and after fracture.

Charpy impact specimens were machined from the 17 mm wall-thickness section. The Charpy fracture samples are shown in Figure 3.

The complete experimental matrix and testing conditions are summarized in Table 2.

### 2.6. Tribological Testing and Three-Dimensional Surface Profilometry

The tribological tests were performed using a ball-on-disk configuration with a 6 mm diameter ball made of 100Cr6 bearing steel. The tests were carried out on disk-shaped specimens with a diameter of 45 mm and a thickness of 8 mm. The tribological specimens were machined from the 48 mm wall-thickness section of the step casting. Prior to testing, the specimen surfaces were ground and polished to achieve a mean surface roughness of R_a_ = 0.64 μm. The surface roughness was measured before testing to ensure comparable initial surface conditions for all specimens. The experiments were conducted under laboratory conditions at a temperature of approximately 20 °C and a relative humidity of 55%. The wear-track cross-sectional area was determined from three-dimensional surface profiles obtained using a Leica DCM 8 confocal optical microscope (Sensofar, Terrassa, Spain).

Tests were conducted under the following conditions:

- Normal load, *FN* = 10 N;
- Sliding distance, *s* = 1000 m;
- Wear-track radius, *r* = 15 mm;
- Dry sliding conditions.

During testing, the friction coefficient (*μ*) and penetration depth were continuously recorded.

Quantitative assessment of wear damage and three-dimensional surface topography was performed using a Leica DCM 8 confocal optical microscope. The following stereometric parameters were determined:

- Maximum wear-track depth (μm);
- Maximum peak height (μm);
- Wear-track cross-sectional area (μm^2^);
- Pile-up area (μm^2^).

These measurements enabled a detailed evaluation of wear mechanisms and the influence of austempering on tribological performance.

## 3. Results

### 3.1. Microstructural Analysis and Hardness

The microstructure of the investigated EN-GJS-800-8 ductile iron was strongly influenced by both the local cooling conditions associated with different wall thicknesses of the cross-step casting (8, 17, 38, and 48 mm) and the applied austempering heat treatment.

#### 3.1.1. As-Cast Condition

In the as-cast condition, distinct variations in matrix constitution were observed as a function of wall thickness. These differences are directly associated with local heat extraction rates and solidification kinetics during casting.

**Figure 4:**
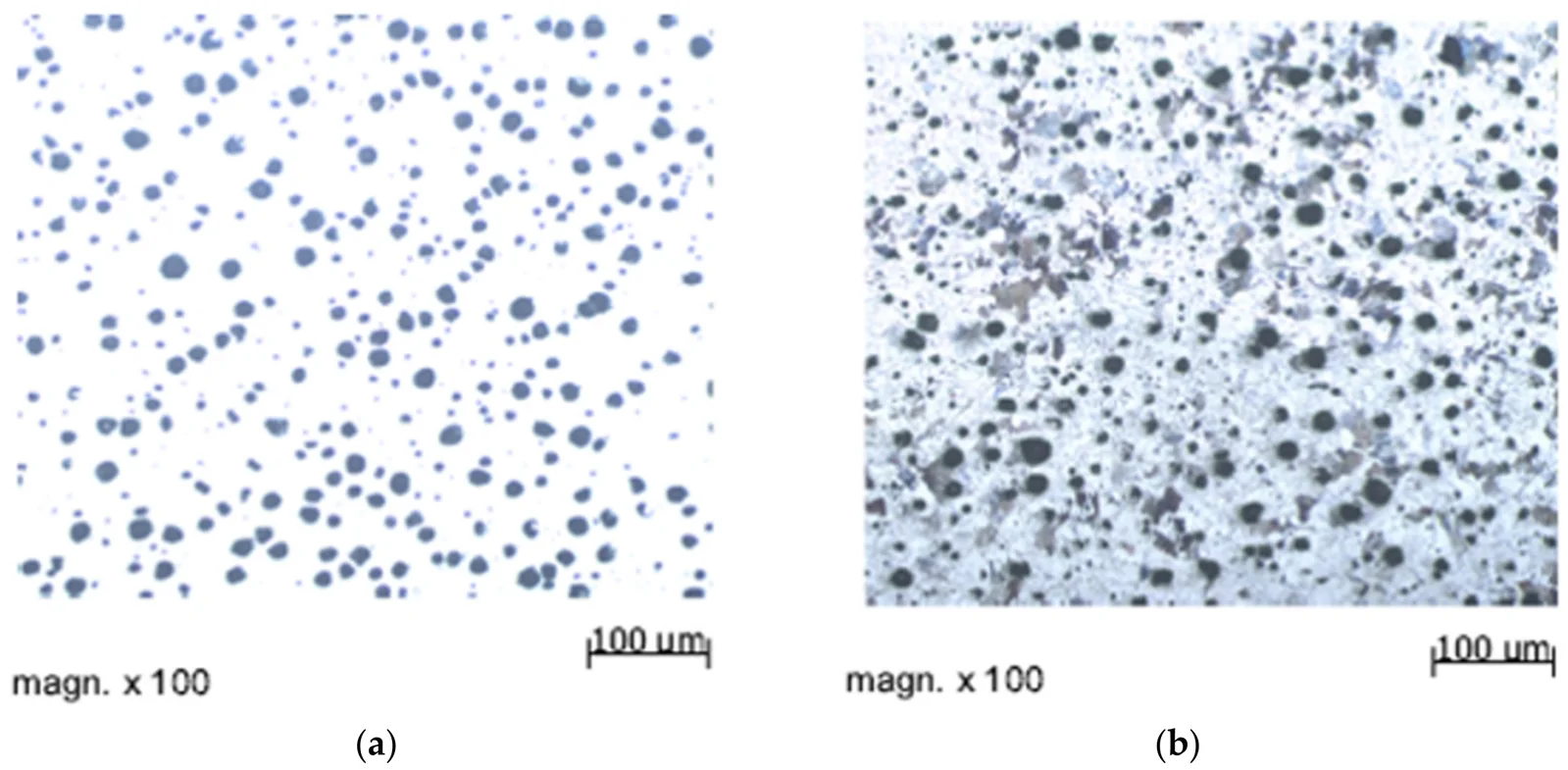
Optical micrographs of EN-GJS-800-8 ductile iron in the as-cast condition, obtained from the 8 mm wall-thickness section: (**a**) polished, unetched specimen used for graphite characterization; (**b**) specimen etched with 3% Nital used for matrix characterization. Magnification: 100×; scale bar: 100 μm.

**Figure 5:**
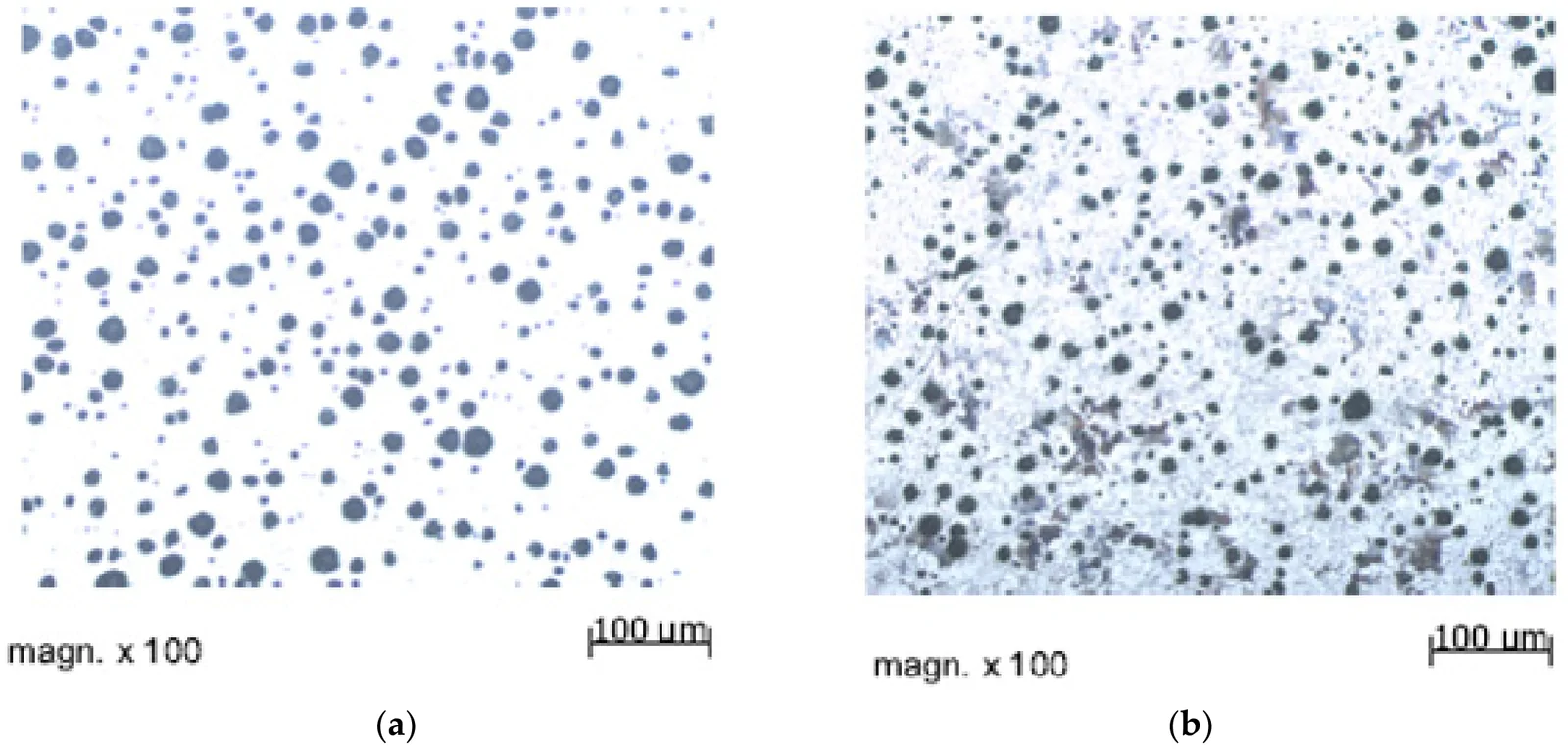
Optical micrographs of EN-GJS-800-8 ductile iron in the as-cast condition, obtained from the 17 mm wall-thickness section: (**a**) polished, unetched specimen used for graphite characterization; (**b**) specimen etched with 3% Nital used for matrix characterization. Magnification: 100×; scale bar: 100 μm.

**Figure 6:**
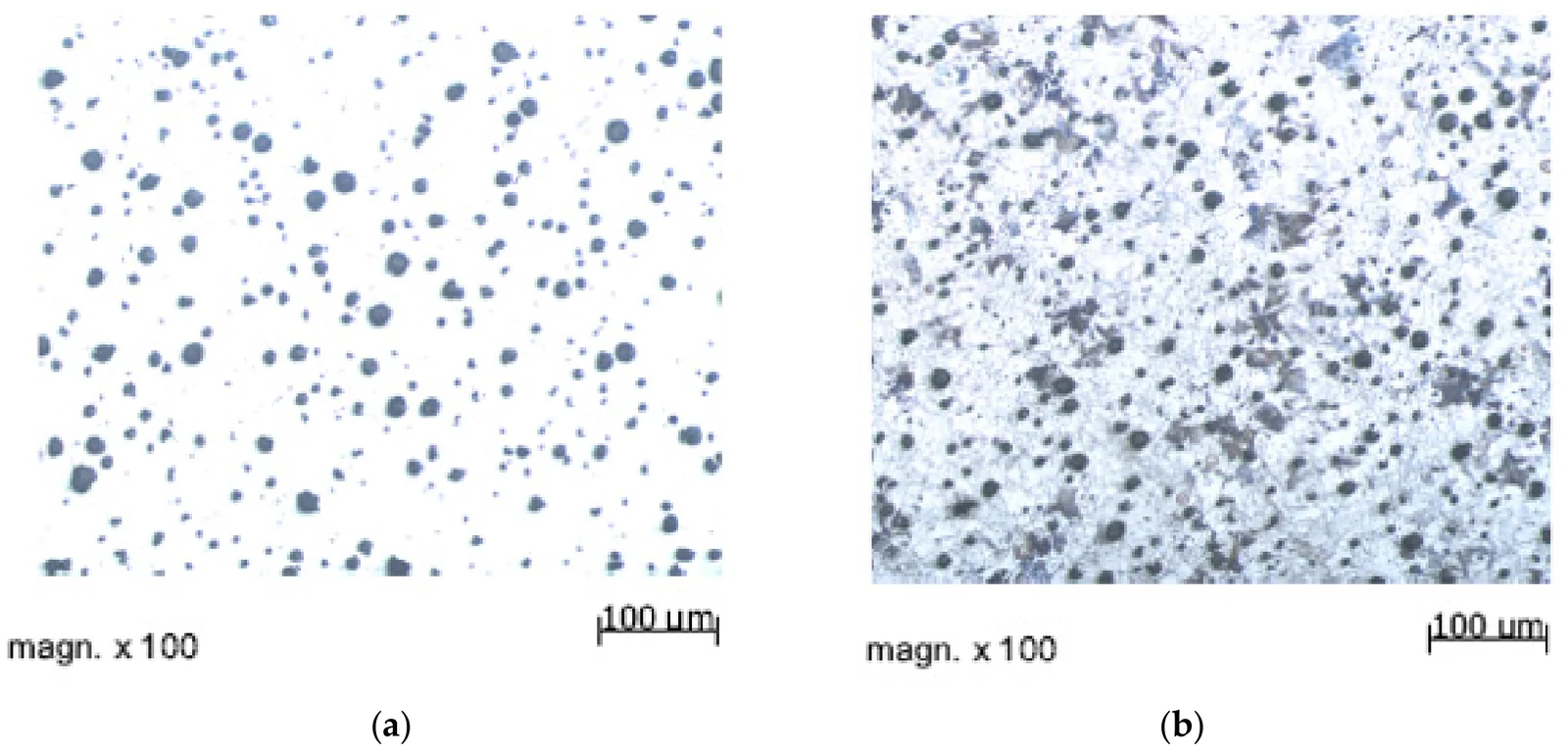
Optical micrographs of EN-GJS-800-8 ductile iron in the as-cast condition, obtained from the 38 mm wall-thickness section: (**a**) polished, unetched specimen used for graphite characterization; (**b**) specimen etched with 3% Nital used for matrix characterization. Magnification: 100×; scale bar: 100 μm.

**Figure 7:**
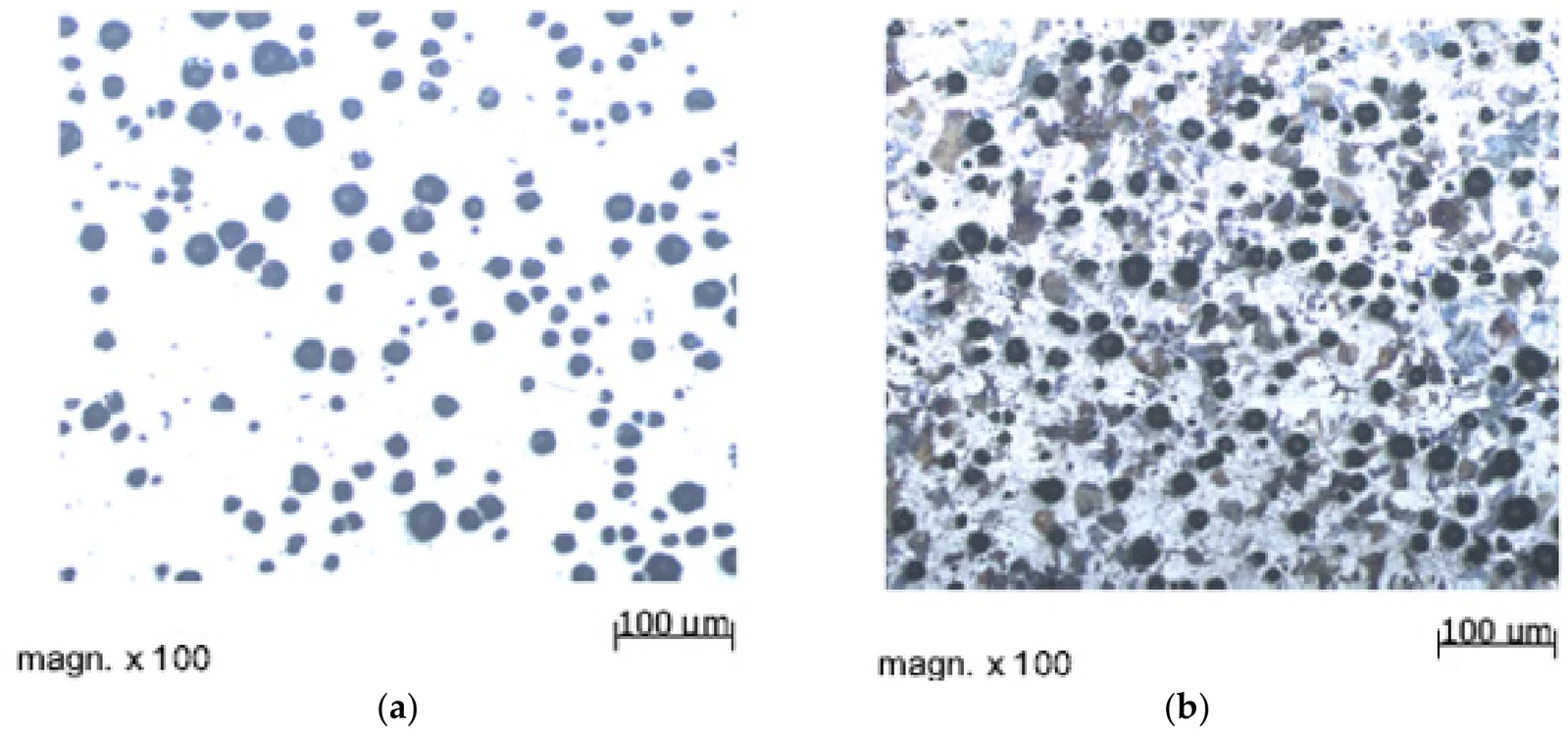
Optical micrographs of EN-GJS-800-8 ductile iron in the as-cast condition, obtained from the 48 mm wall-thickness section: (**a**) polished, unetched specimen used for graphite characterization; (**b**) specimen etched with 3% Nital used for matrix characterization. Magnification: 100×; scale bar: 100 μm.

The thinnest section (8 mm), which experienced the highest cooling rate, contained the highest pearlite fraction (35.2%) and the largest graphite nodule density (326 mm^2^). In contrast, the 48 mm section contained 95.0% ferrite and only 141 graphite nodules mm^2^. These trends are consistent with slower solidification and enhanced carbon diffusion in thicker sections. The as-cast hardness values did not decrease monotonically with increasing ferrite content; therefore, the measured hardness should not be attributed to the ferrite-to-pearlite ratio alone, because local segregation, graphite characteristics, and sampling location may also contribute. Representative micrographs are shown in Figure 4, Figure 5, Figure 6 and Figure 7.

The 8 mm section exhibits the highest graphite nodule density (326 mm^2^) and the largest pearlite fraction (35.2%) among the as-cast sections. The fine graphite dispersion visible in Figure 4a and the mixed ferritic–pearlitic matrix in Figure 4b correspond to 64.8% ferrite and an average hardness of 190.6 ± 2 HBW reported in Table 3.

The 17 mm section retains a high graphite nodule density of 303 mm^2^ and complete nodularity (100.0%), while the graphite area fraction reaches 17.4%. The predominantly ferritic matrix visible in Figure 5b is consistent with 87.3% ferrite, 12.7% pearlite, and an average hardness of 200.0 ± 2.2 HBW in Table 3.

The 38 mm section shows a lower graphite nodule density (263 mm^2^) than the thinner sections and the lowest measured graphite area fraction (8.9%). The matrix observed in Figure 6b is predominantly ferritic, corresponding to 89.6% ferrite, 10.4% pearlite, and an average hardness of 207.0 ± 1 HBW in Table 3.

The 48 mm section exhibits the lowest graphite nodule density (141 mm^2^) and the most ferritic matrix among the as-cast sections. The more widely spaced graphite nodules and the matrix shown in Figure 7 correspond to 100.0% nodularity, 95.0% ferrite, 5.0% pearlite, and an average hardness of 207.8 ± 1.5 HBW in Table 3.

Quantitative metallography confirmed that graphite nodule density decreased systematically from 326 mm^2^ in the 8 mm section to 141 mm^2^ in the 48 mm section (Table 4). Nodularity remained very high (99.6–100.0%), indicating that wall thickness affected nodule density more strongly than graphite shape. The graphite area fraction varied non-monotonically between 8.9% and 17.4%, showing that this parameter was not controlled by section thickness alone.

The ferrite fraction increased from 64.8% in the 8 mm section to 95.0% in the 48 mm section, while the pearlite fraction decreased from 35.2% to 5.0%. The combined changes in matrix constitution and nodule density demonstrate the strong influence of local solidification conditions on the as-cast microstructure.

#### 3.1.2. Austempered Condition

After austenitization at 880–890 °C for 2.5 h and austempering at 370–380 °C for 2.0 h, the initially heterogeneous ferritic–pearlitic matrix was replaced in all sections by a fine acicular morphology that was optically more uniform than the as-cast matrix.

**Figure 8:**
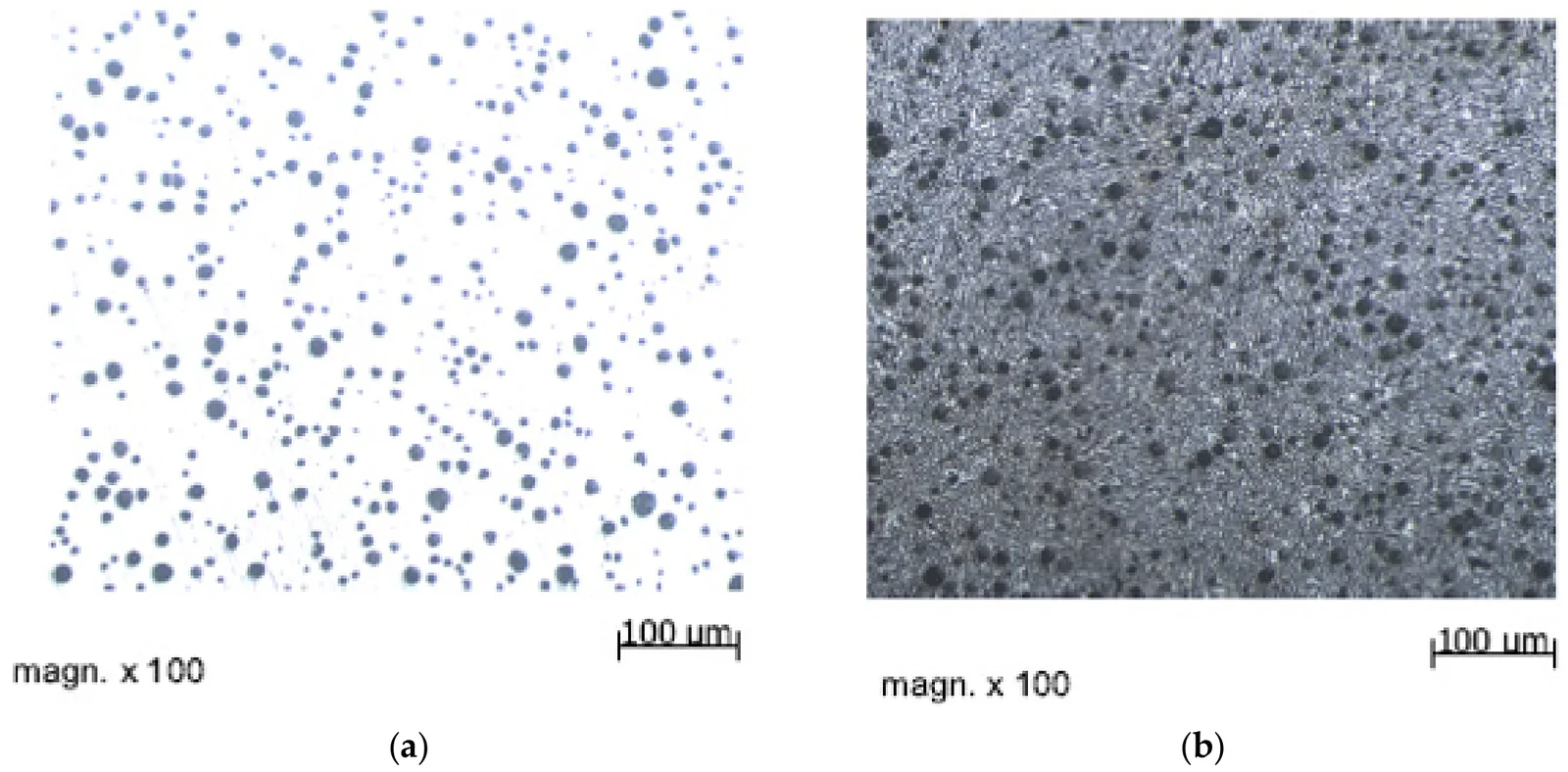
Optical micrographs of EN-GJS-800-8 ductile iron in the austempered condition, obtained from the 8 mm wall-thickness section: (**a**) polished, unetched specimen used for graphite characterization; (**b**) specimen etched with 3% Nital used for matrix characterization. Magnification: 100×; scale bar: 100 μm.

**Figure 9:**
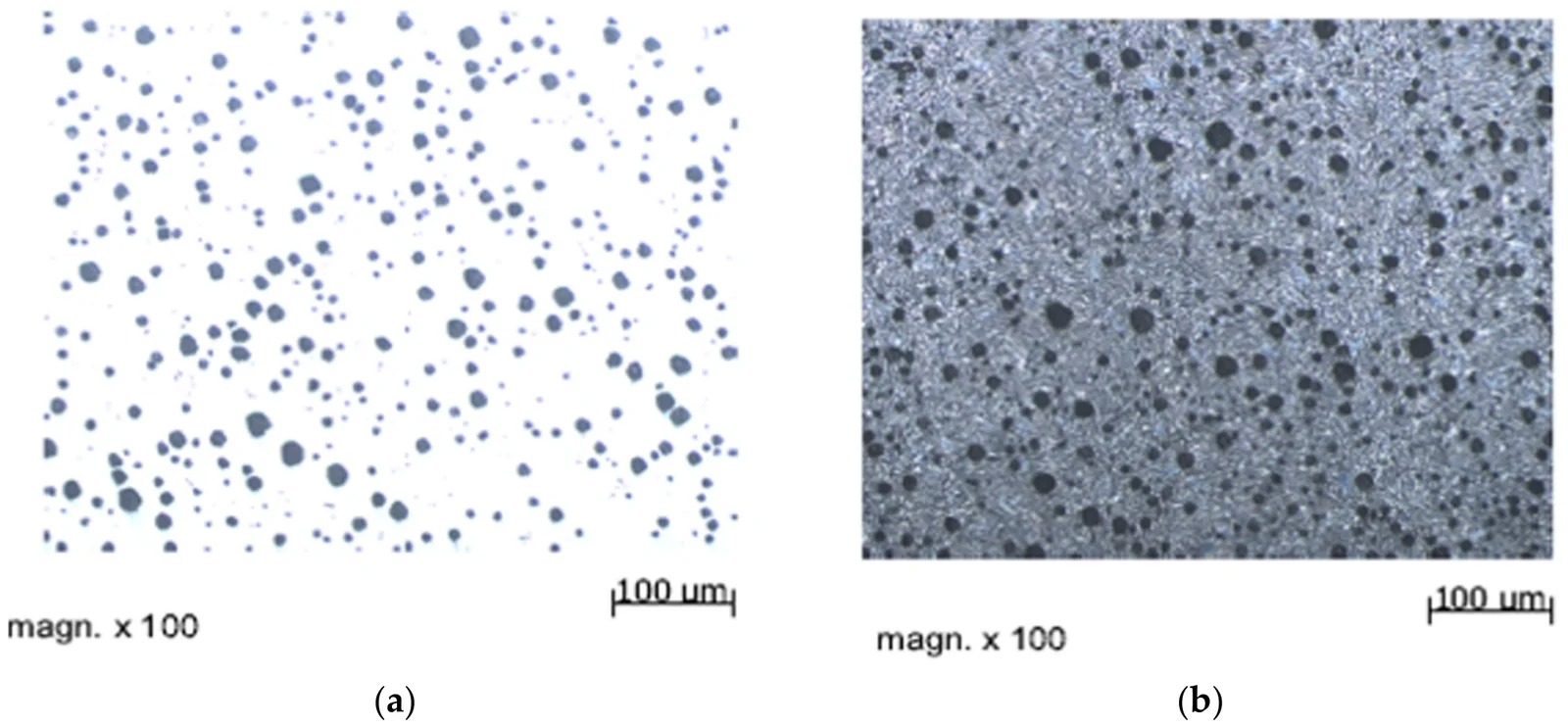
Optical micrographs of EN-GJS-800-8 ductile iron in the austempered condition, obtained from the 17 mm wall-thickness section: (**a**) polished, unetched specimen used for graphite characterization; (**b**) specimen etched with 3% Nital used for matrix characterization. Magnification: 100×; scale bar: 100 μm.

**Figure 10:**
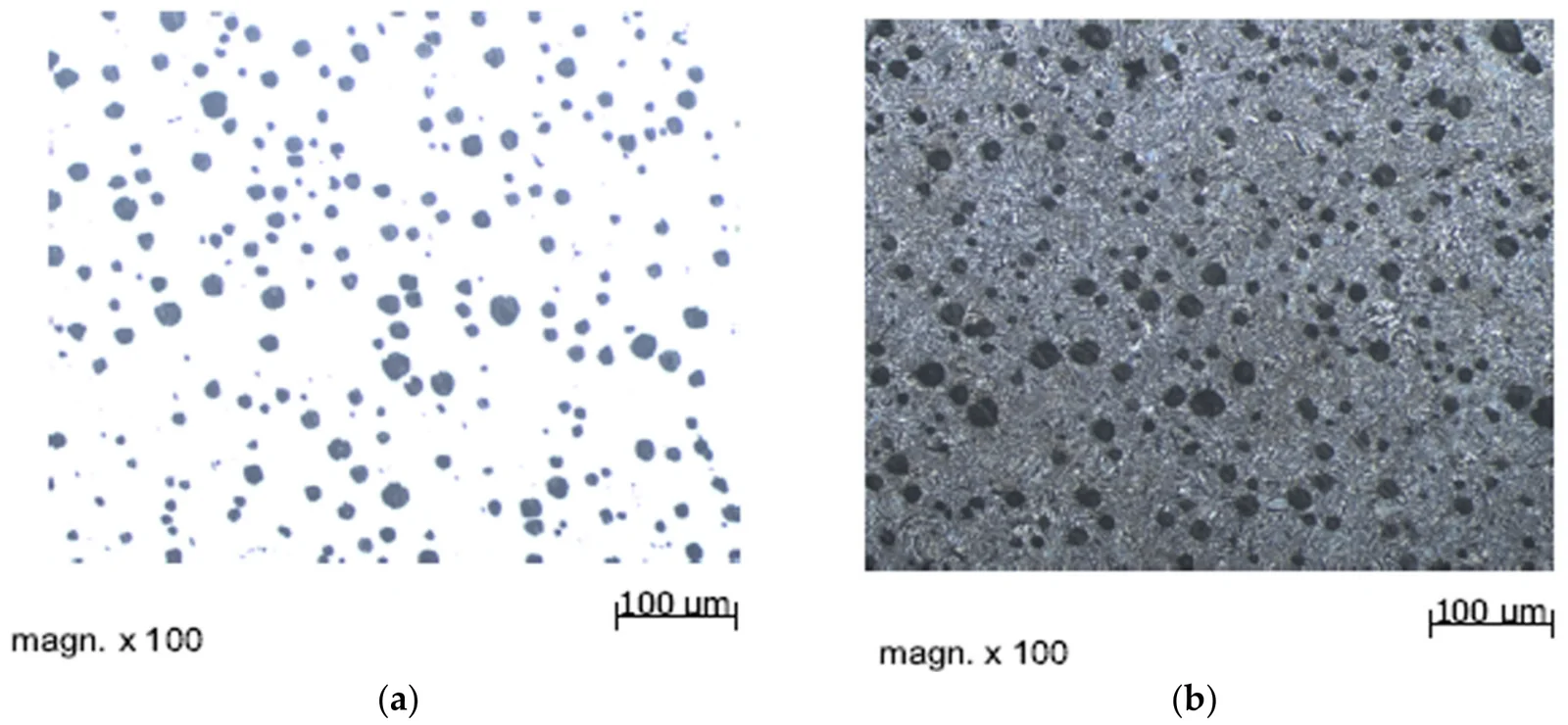
Optical micrographs of EN-GJS-800-8 ductile iron in the austempered condition, obtained from the 38 mm wall-thickness section: (**a**) polished, unetched specimen used for graphite characterization; (**b**) specimen etched with 3% Nital used for matrix characterization. Magnification: 100×; scale bar: 100 μm.

**Figure 11:**
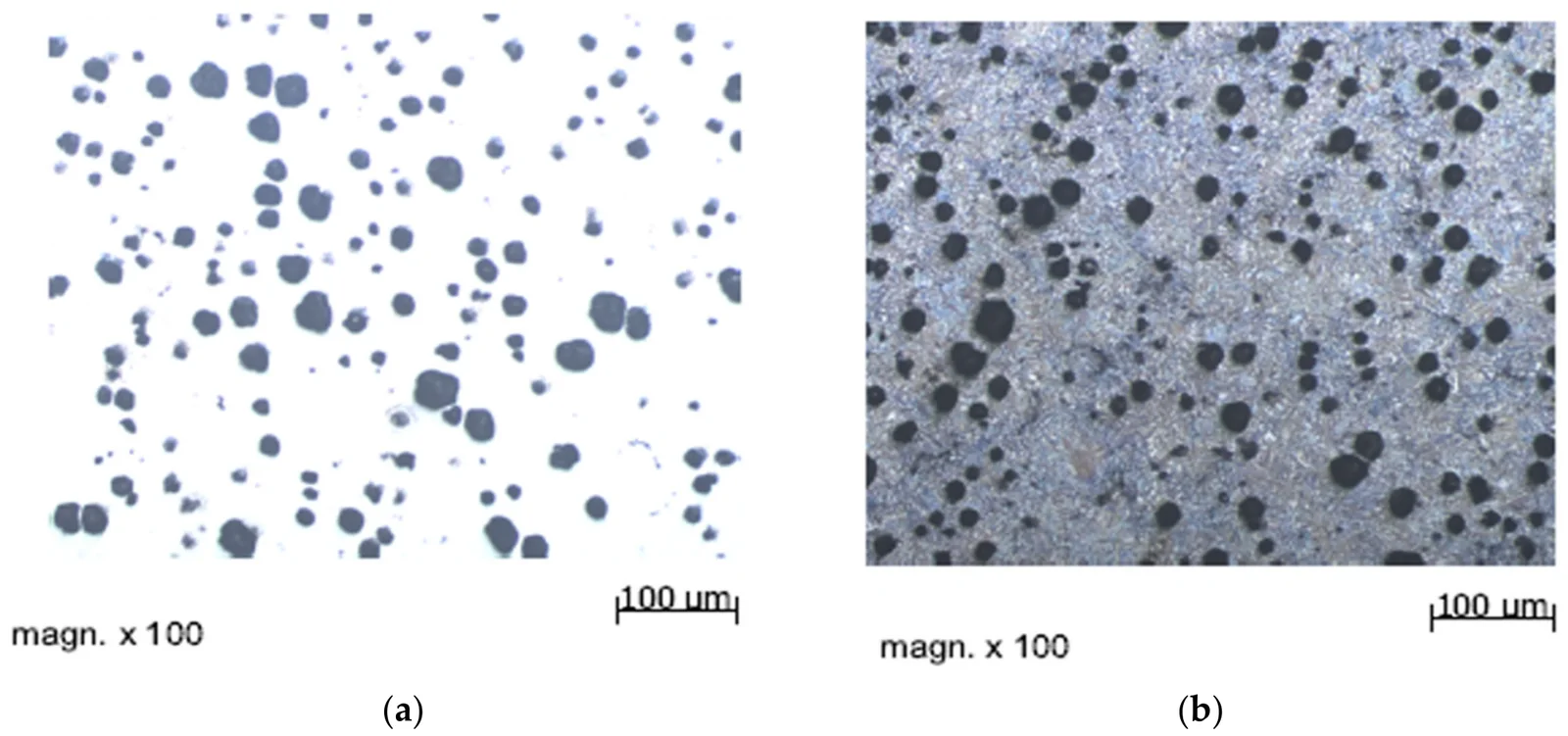
Optical micrographs of EN-GJS-800-8 ductile iron in the austempered condition, obtained from the 48 mm wall-thickness section: (**a**) polished, unetched specimen used for graphite characterization; (**b**) specimen etched with 3% Nital used for matrix characterization. Magnification: 100×; scale bar: 100 μm.

The etched micrographs were consistent with an ausferritic matrix. This identification is based on the observed morphology and the applied heat-treatment path; optical microscopy alone cannot quantify retained austenite or exclude small fractions of other phases. Representative micrographs are shown in Figure 8, Figure 9, Figure 10 and Figure 11.

After austempering, the 8 mm section displays a fine, optically uniform acicular matrix consistent with ausferrite, while the graphite remains highly nodular. Table 5 reports the highest graphite nodule density (379 mm^2^), 100.0% nodularity, a graphite area fraction of 9.9%, and the highest average hardness of 300.4 ± 1.8 HBW.

The 17 mm section also exhibits a uniform acicular matrix and well-dispersed spheroidal graphite after austempering. These observations correspond to 337 graphite nodules mm^2^, 100.0% nodularity, an 11.2% graphite area fraction, and an average hardness of 298.0 ± 1.6 HBW in Table 5.

In the 38 mm section, the graphite nodule density decreases to 224 mm^2^, although nodularity remains high at 99.1%. The etched micrograph shows an optically homogeneous ausferritic matrix, consistent with the 13.1% graphite area fraction and average hardness of 299.6 ± 1.3 HBW reported in Table 5.

The 48 mm section contains the lowest graphite nodule density after austempering (147 mm^2^) and the highest graphite area fraction (19.9%). Despite the coarser graphite distribution, the matrix remains optically uniform and ausferritic; Table 5 reports 100.0% nodularity and an average hardness of 287.6 ± 2 HBW.

The similar acicular morphology observed in all four sections, together with the narrower range of hardness after heat treatment, indicates that the selected cycle substantially reduced the influence of the initial wall-thickness-dependent microstructure on the final material response.

Direct confirmation of the retained-austenite fraction and any minor transformation products would require phase-sensitive methods such as X-ray diffraction (XRD) or electron backscatter diffraction (EBSD), which were outside the scope of the present study.

#### 3.1.3. Hardness Evolution

The microstructural transformation induced by austempering was accompanied by a substantial increase in hardness. Average Brinell hardness values increased from 190.6 to 207.8 HBW in the as-cast condition to 287.6–300.4 HBW after heat treatment (Table 3).

A comparison of the 95% confidence intervals revealed that the differences in hardness between individual wall-thickness sections became significantly smaller after austempering. This observation indicates that the heat-treatment process effectively reduced the influence of local solidification conditions on the final mechanical response of the material.

The highest hardness value after austempering was recorded for the 8 mm section (300.4 ± 1.82 HBW), whereas the lowest value was measured for the 48 mm section (287.6 ± 1.95 HBW). Despite these minor differences, all sections exhibited hardness levels characteristic of fully austempered ductile iron.

The increase in hardness can be attributed to the replacement of the ferritic–pearlitic matrix by a considerably stronger ausferritic structure composed of acicular ferrite and carbon-stabilized retained austenite. Similar hardness levels have been reported in previous studies on upper-ausferrite ADI produced at austempering temperatures between 350 and 400 °C [10,15,18].

The quantitative microstructural parameters obtained for both material conditions are summarized in Table 4 and Table 5, while the hardness results together with statistical analysis are presented in Table 3.

**Table 3:** Brinell hardness results and statistical analysis (*n* = 5).

Wall Thickness (mm)	As-Cast Condition—Mean Hardness (HBW)	As-Cast Condition—95% CI (HBW)	ADI Condition—Mean Hardness (HBW)	ADI Condition—95% CI (HBW)
**8**	190.6 ± 2	188.2–193	300.4 ± 1.8	298.1–302.7
**17**	200.0 ± 2.2	197.2–202.8	298.0 ± 1.6	296–300
**38**	207 ± 1	205.8–208.2	299.6 ± 1.34	297.9–301.3
**48**	207.8 ± 1.5	206–209.6	287.6 ± 1.95	285.2–290

**Table 4:** Quantitative microstructural parameters of EN-GJS-800-8 ductile iron in the as-cast condition.

Wall Thickness (mm)	Graphite Nodule Count [1/mm^2^]	Nodularity (%)	Graphite Area Fraction (%)	Ferrite Fraction in the Matrix (%)	Pearlite Fraction in the Matrix (%)	Average Hardness (HBW)
**8**	326	99.7	14.6	64.8	35.2	190.6 ± 2
**17**	303	100.0	17.4	87.3	12.7	200.0 ± 2.2
**38**	263	99.6	8.9	89.6	10.4	207.0 ± 1
**48**	141	100	15.9	95.0	5.0	207.8 ± 1.5

**Table 5:** Quantitative microstructural parameters of EN-GJS-800-8 ductile iron after austempering (ADI condition).

Wall Thickness (mm)	Graphite Nodule Count [1/mm^2^]	Nodularity (%)	Graphite area Fraction (%)	Matrix Type	Average Hardness (HBW)
8	379	100.0	9.9	Ausferritic	300.4 ± 1.8
17	337	100.0	11.2	Ausferritic	298 ± 1.6
38	224	99.1	13.1	Ausferritic	299.6 ± 1.3
48	147	100.0	19.9	Ausferritic	287.6 ± 2

#### 3.1.4. Quantitative Relationships Between Wall Thickness, Microstructure, and Hardness

In the as-cast condition, increasing wall thickness was associated with a decrease in graphite nodule density from 326 to 141 mm^−2^, an increase in ferrite fraction from 64.8% to 95.0%, and a decrease in pearlite fraction from 35.2% to 5.0%. These results quantify the effect of reduced cooling rate on the microstructure of the cross-step casting.

Hardness in the as-cast condition ranged from 190.6 to 207.8 HBW but did not follow the ferrite fraction monotonically. Accordingly, the hardness response reflects the combined influence of matrix constitution, graphite distribution, local chemical heterogeneity, and measurement location rather than a single microstructural parameter.

After austempering, the hardness range shifted to 287.6–300.4 HBW and the etched matrix displayed a similar acicular morphology in all wall-thickness sections. This result indicates that the selected treatment increased hardness while reducing the sensitivity of the final response to the initial casting geometry.

The quantitative data therefore support two distinct conclusions: wall thickness strongly affected the as-cast graphite and matrix parameters, whereas austempering promoted a substantially more uniform microstructural and mechanical response. Direct correlations with retained-austenite content cannot be established without phase-sensitive measurements.

### 3.2. Mechanical Properties and Microstructural Interpretation

The improvement in mechanical performance after austempering is consistent with the replacement of the ferritic–pearlitic matrix by a fine acicular morphology characteristic of ADI. The selected temperature range of 370–380 °C corresponds to the upper-ausferrite transformation region reported in the literature.

During isothermal holding, carbon rejected from growing ferrite plates enriches the surrounding austenite and may stabilize it to room temperature. This established mechanism explains the favorable strength–ductility balance commonly reported for ADI [10,15,18]; however, the retained-austenite fraction was not measured directly in the present study.

#### 3.2.1. Tensile Properties

The tensile-test results revealed a significant enhancement of mechanical performance after austempering. The ultimate tensile strength (R_m_) increased from 567 MPa in the as-cast condition to 935 MPa after heat treatment, corresponding to an increase of approximately 65%. Simultaneously, the yield strength (R_p0.2_) increased from 418 MPa to 592 MPa.

**Figure 12:**
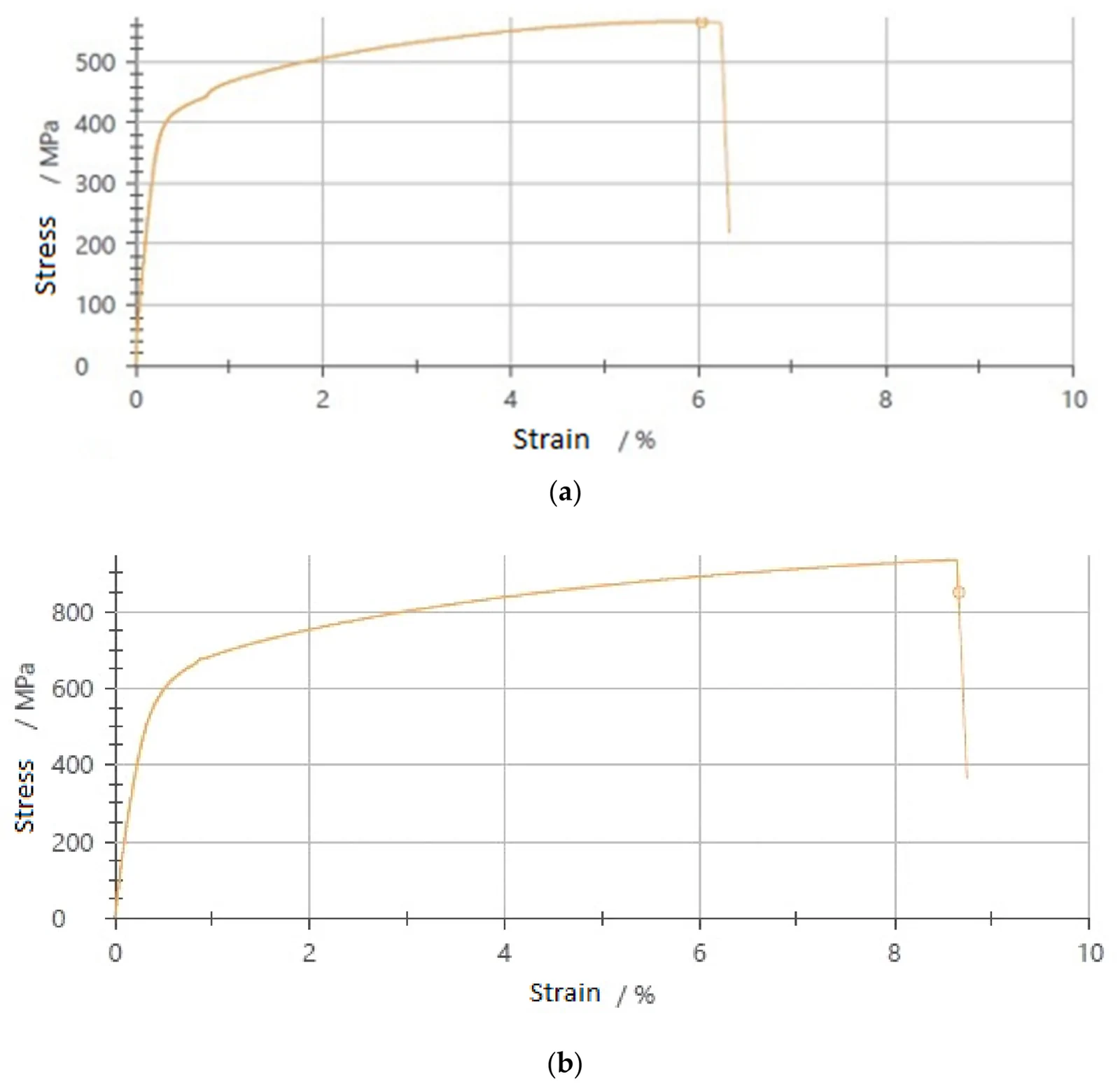
Engineering stress–strain curves obtained for EN-GJS-800-8 ductile iron in the (**a**) as-cast and (**b**) austempered conditions.

The stress–strain curves obtained for both material conditions are presented in Figure 12.

The increase in strength can be attributed primarily to the formation of the ausferritic matrix, which possesses a considerably higher resistance to plastic deformation than the original ferritic–pearlitic structure. The fine dispersion of acicular ferrite contributes to strengthening by impeding dislocation motion, while retained austenite provides additional strain accommodation during deformation.

An important observation is that the increase in strength was accompanied by an improvement in elongation from 6.2% to 8.6%. Such a combination of strength and ductility is one of the principal advantages of austempered ductile iron and distinguishes ADI from many conventionally hardened ferrous alloys.

#### 3.2.2. Interpretation of the Possible TRIP Contribution

The simultaneous increase in strength and elongation may indicate a beneficial contribution of retained austenite during plastic deformation. Similar behavior has frequently been associated in the literature with the Transformation-Induced Plasticity (TRIP) mechanism, whereby metastable retained austenite progressively transforms into martensite under applied stress or strain [9,16,24].

However, it should be emphasized that the present study did not include direct quantitative measurements of retained austenite content using X-ray diffraction (XRD), electron backscatter diffraction (EBSD), or magnetic characterization techniques. Therefore, the occurrence of the TRIP effect cannot be experimentally confirmed within the scope of this work.

Consequently, the role of TRIP should be regarded as a literature-supported interpretation of the observed mechanical behavior rather than as a directly verified transformation mechanism. Nevertheless, the measured combination of increased strength and improved ductility is consistent with trends commonly reported for ADI grades containing stabilized retained austenite [9,10].

#### 3.2.3. Impact Toughness

Charpy V-notch testing demonstrated a substantial increase in absorbed impact energy following austempering. The measured impact energies increased from 4.60 to 5.60 J in the as-cast condition to 10.01–10.11 J after heat treatment. Table 6 shows the results of the Charpy test in the as-cast and after hardening.

The mean absorbed impact energy increased from 5.12 J in the as-cast condition to 10.06 J after austempering, corresponding to an increase of approximately 96.5%. The result demonstrates improved impact-energy absorption after formation of the acicular transformed matrix.

From a microstructural perspective, retained austenite may contribute to energy dissipation through localized plastic deformation ahead of the crack tip. In ADI materials, such behavior is frequently associated with stress-assisted transformation processes and local strain accommodation mechanisms reported in the literature [9,10]. However, as discussed previously, confirmation of these mechanisms would require dedicated phase-identification studies.

Overall, the impact-test results demonstrate that the selected austempering parameters not only increased hardness and tensile strength but also improved Charpy impact energy, leading to a more balanced combination of mechanical properties.

#### 3.2.4. Relationship Between Microstructure and Mechanical Performance

The obtained results clearly demonstrate that the transformation from a ferritic–pearlitic matrix to a homogeneous ausferritic structure fundamentally alters the mechanical response of EN-GJS-800-8 ductile iron.

The increase in hardness, tensile strength, elongation, and impact energy indicates that the selected heat-treatment parameters successfully produced a microstructure capable of simultaneously providing strength, ductility, and toughness. Such a combination of properties is particularly attractive for engineering components subjected to complex loading conditions involving static, cyclic, and impact stresses.

Furthermore, the relatively uniform hardness values measured across all wall-thickness sections indicate that the selected heat-treatment cycle effectively minimized the influence of casting-section geometry on the final mechanical performance. This finding is particularly important for industrial applications involving castings with variable wall thicknesses, where property homogeneity is often a critical requirement.

### 3.3. Tribological Properties and Abrasive Wear Resistance

**Figure 13:**
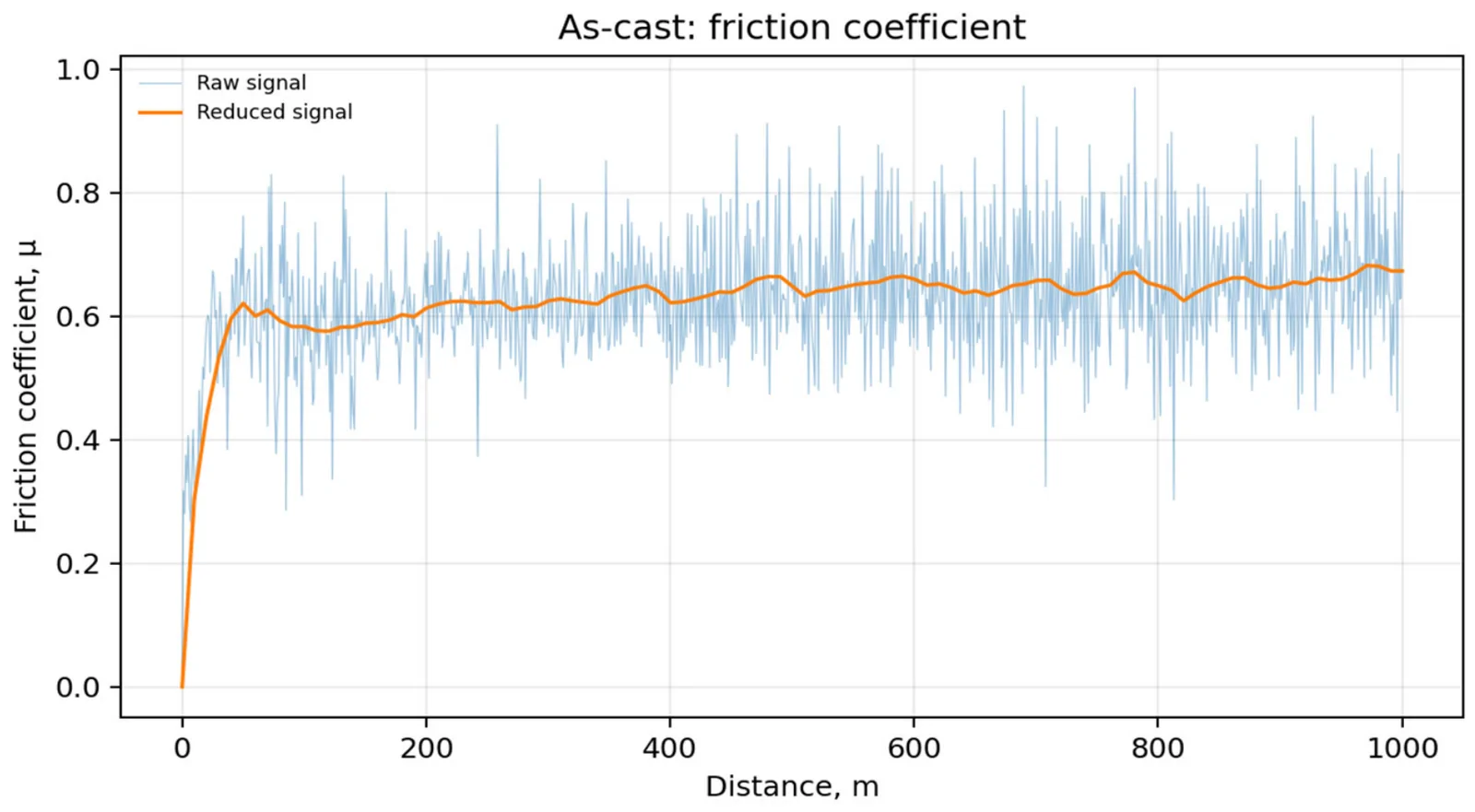

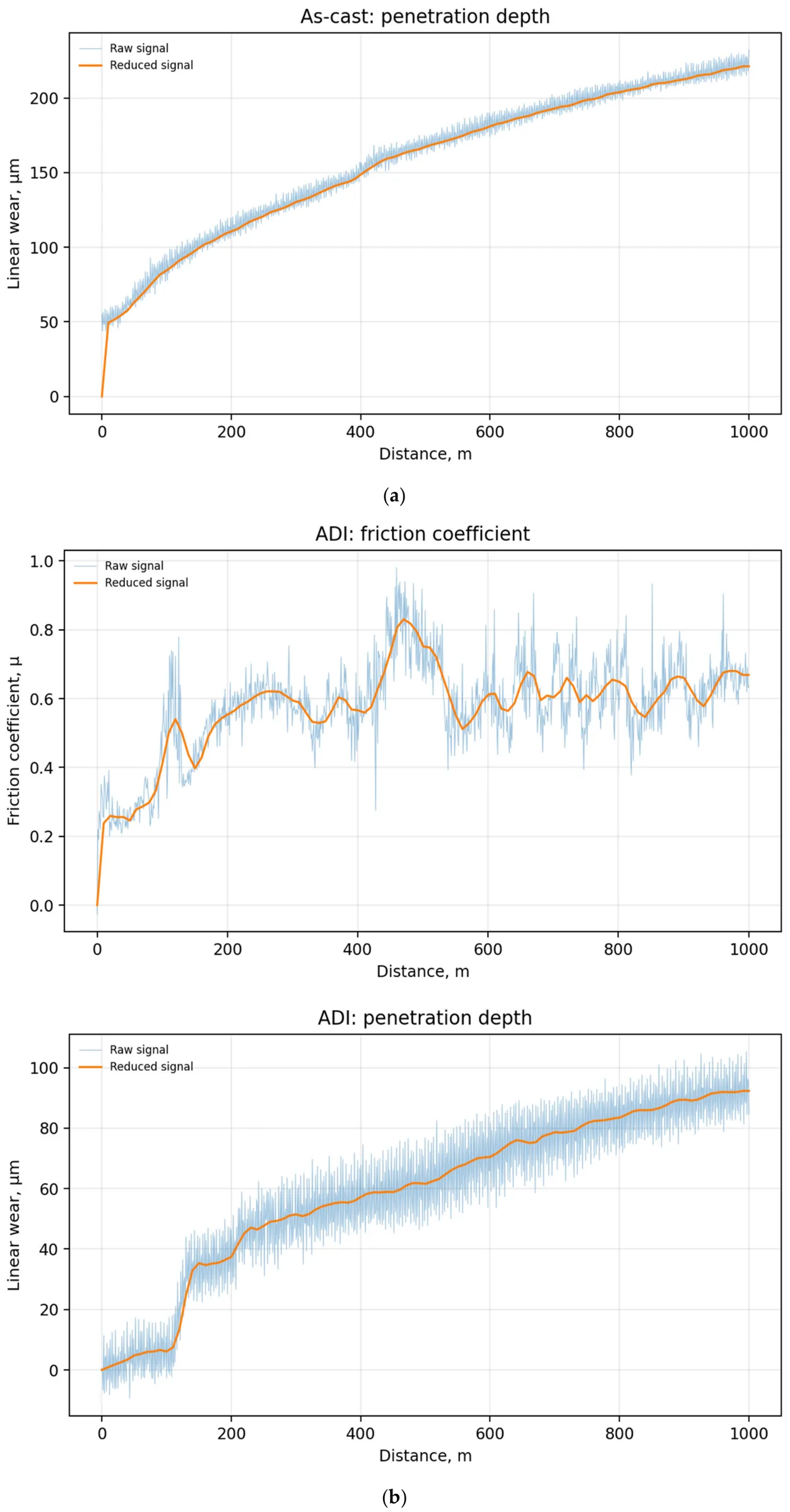
Evolution of the friction coefficient and penetration depth during dry sliding wear tests: (**a**) as-cast condition and (**b**) austempered ADI condition.

The implementation of the ausferritic matrix significantly altered the tribological response of the investigated EN-GJS-800-8 ductile iron under dry sliding conditions. The evolution of the friction coefficient (*μ*) and penetration depth as a function of sliding distance for both the as-cast and austempered conditions is presented in Figure 13a,b.

#### 3.3.1. Friction Behavior

In the as-cast condition, the average friction coefficient stabilized at approximately *μ* ≈ 0.65. However, significant high-frequency fluctuations were observed, with instantaneous values approaching 0.90. Such behavior is characteristic of repeated adhesion and rupture events occurring at asperity contacts within the ferritic–pearlitic matrix.

The continuous oscillations of the friction coefficient indicate unstable contact conditions and progressive degradation of the surface layer during sliding.

Following austempering, the average friction coefficient remained at a comparable level (*μ* ≈ 0.63). Although macroscopic fluctuations were still observed, the overall friction behavior became more stable with respect to wear progression. Similar friction characteristics have been reported for ausferritic ductile irons operating under dry sliding conditions [9,14].

The relatively small difference in average friction coefficient between the two material conditions suggests that the improvement in tribological performance is not primarily associated with friction reduction but rather with a substantial enhancement in wear resistance.

#### 3.3.2. Wear Resistance

The most significant effect of austempering was observed in the wear behavior of the material.

In the as-cast condition, the penetration depth increased continuously throughout the test, reaching approximately 225 μm after a sliding distance of 1000 m. This behavior indicates progressive material removal and limited resistance of the ferritic–pearlitic matrix to concentrated contact stresses.

In contrast, the austempered specimens exhibited dramatically lower wear rates. The maximum penetration depth recorded at the end of the test did not exceed 95 μm, representing a reduction of nearly 60% compared with the as-cast condition.

These results demonstrate the superior load-bearing capacity and structural stability of the ausferritic matrix under dry sliding conditions.

#### 3.3.3. Three-Dimensional Wear-Track Analysis

Direct confirmation of the enhanced wear resistance was obtained through three-dimensional surface profilometry performed using a Leica DCM 8 confocal microscope.

**Figure 14:**
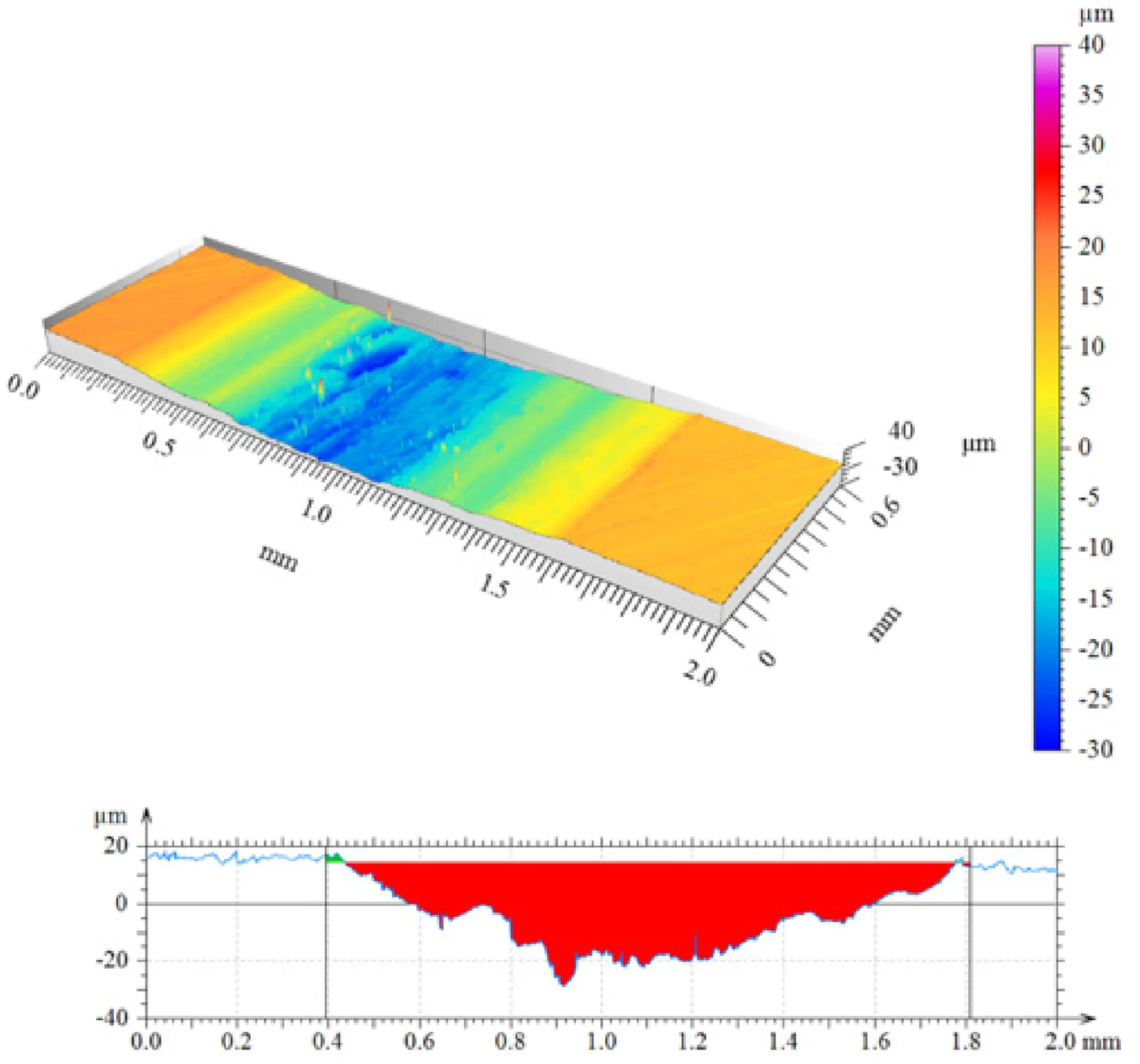
Three-dimensional wear-track morphology of EN-GJS-800-8 ductile iron in the as-cast condition.

**Figure 15:**
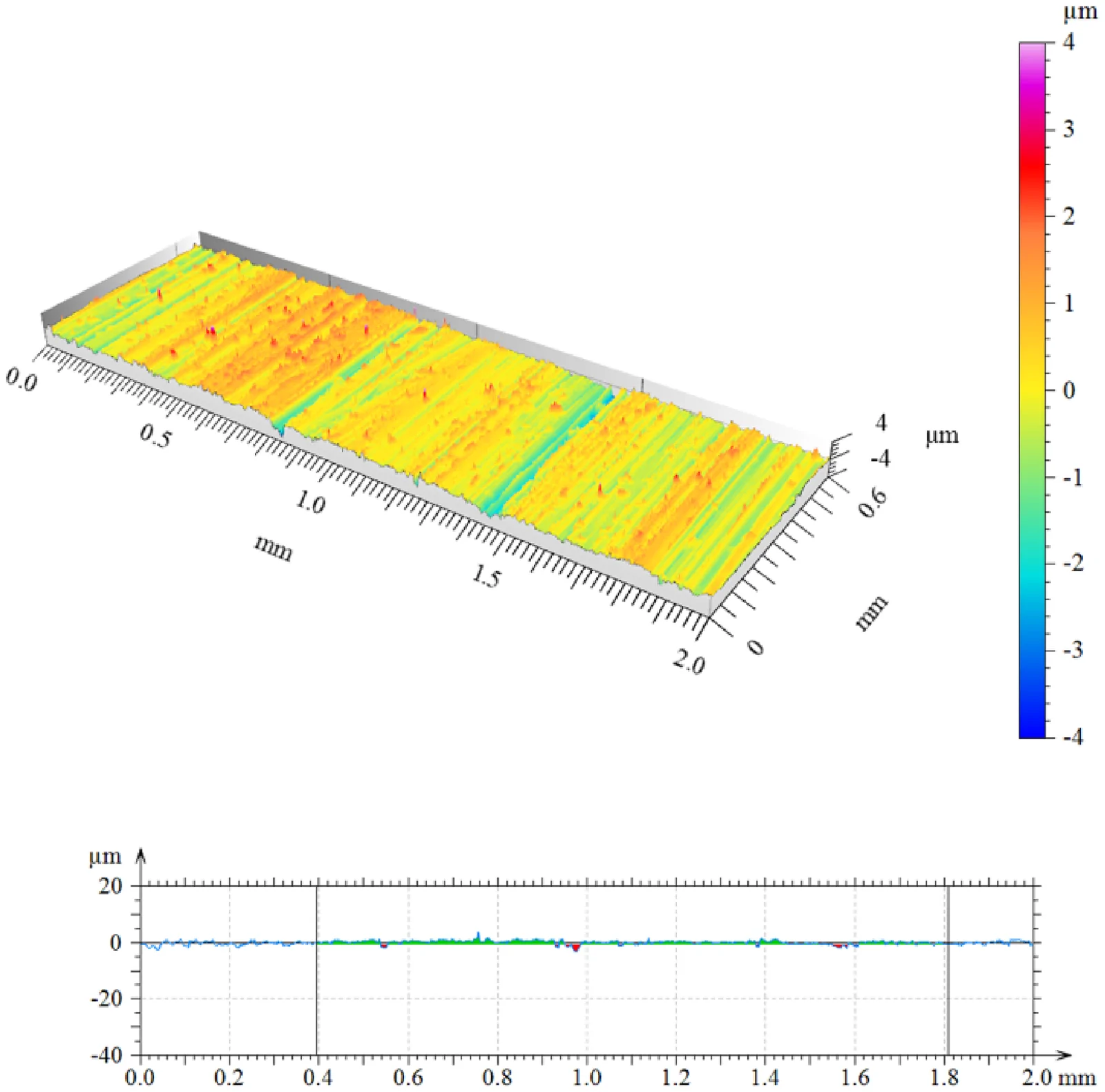
Three-dimensional wear-track morphology of EN-GJS-800-8 ductile iron after austempering.

Representative wear tracks obtained for the as-cast and austempered conditions are shown in Figure 14 and Figure 15.

The quantitative stereometric parameters extracted from the wear tracks are summarized in Table 7.

The maximum wear-track depth decreased from 43.5 μm in the as-cast condition to only 3 μm after austempering, corresponding to a reduction exceeding 93%.

An even more pronounced effect was observed for the wear-track cross-sectional area, which decreased from 2.98 × 10^4^ μm^2^ to 210 μm^2^. This corresponds to more than a 140-fold reduction in material loss, clearly demonstrating the remarkable effectiveness of austempering in improving abrasive wear resistance.

Interestingly, the maximum peak height increased from 2.8 μm to 4.1 μm after heat treatment, while the pile-up area increased from 71.4 μm^2^ to 673.8 μm^2^. These features indicate enhanced local plastic deformation at the edges of the wear track and suggest a greater ability of the surface layer to accommodate contact stresses without extensive material removal.

#### 3.3.4. Wear Mechanisms

The wear mechanisms differed substantially between the two investigated material conditions.

In the as-cast state, three-dimensional surface analysis revealed deep grooves and extensive plowing marks characteristic of abrasive micro-cutting. The relatively soft ferritic regions were particularly susceptible to penetration by hard asperities, leading to significant material displacement and removal. The resulting wear morphology is consistent with abrasive wear mechanisms reported for ductile and austempered ductile irons under dry-sliding conditions [4,17].

In contrast, the austempered material exhibited a very shallow wear track accompanied by pronounced pile-up regions at the track boundaries. The significantly lower penetration depth indicates that the ausferritic matrix effectively resisted localized deformation induced by the tribological contact.

The enhanced wear resistance may be attributed to several complementary mechanisms. First, the inherently higher hardness of the ausferritic matrix reduces the susceptibility of the material to abrasive penetration. Second, the presence of carbon-enriched retained austenite may contribute to local strain accommodation under contact loading. Finally, previous studies have reported that deformation-induced transformation of retained austenite can locally increase surface hardness during wear, thereby improving resistance to further material removal [16,17,24].

It should be emphasized, however, that the present work did not include direct phase-transformation measurements during tribological testing. Consequently, any contribution of deformation-induced martensitic transformation should be regarded as a plausible literature-supported interpretation rather than a mechanism experimentally verified within this study.

The substantial reduction in wear-track depth and cross-sectional area after austempering is consistent with the simultaneous increase in hardness and the formation of a homogeneous ausferritic matrix. These results demonstrate a clear correlation between the microstructural evolution induced by austempering and the tribological performance of the investigated ductile iron. The enhanced wear resistance can therefore be attributed primarily to the improved load-bearing capacity and resistance to plastic deformation of the ausferritic matrix.

#### 3.3.5. Engineering Implications

From an engineering perspective, the observed increase in wear resistance is highly significant. The combination of high tensile strength, improved toughness, and exceptional resistance to abrasive wear makes austempered EN-GJS-800-8 ductile iron an attractive candidate for components operating under severe contact and sliding conditions.

Potential applications include gears, sprockets, mining equipment components, agricultural machinery parts, railway systems, and other heavily loaded mechanical assemblies where wear resistance and structural reliability are critical design requirements.

The more than 140-fold reduction in wear-track cross-sectional area achieved through austempering demonstrates the substantial performance gains that can be obtained through appropriate microstructural engineering of ductile iron castings.

### 3.4. Discussion

The present investigation demonstrates that austempering is an effective route for improving the engineering performance of EN-GJS-800-8 ductile iron produced as cross-step castings with variable wall thickness.

The as-cast material exhibited clear microstructural heterogeneity caused by differences in local cooling rate. Thinner sections contained more pearlite and a higher graphite nodule density, whereas thicker sections were predominantly ferritic and contained fewer nodules. These observations agree with the established influence of section thickness on ductile-iron solidification [6,8,11,12,13].

Following austempering, the ferritic–pearlitic matrix was replaced by an optically uniform acicular morphology in all investigated sections. The narrower hardness range after heat treatment shows that the selected cycle substantially reduced the influence of casting geometry on the final response.

Mechanical testing showed simultaneous increases in hardness, ultimate tensile strength, elongation, and Charpy impact energy. This combination is consistent with the strengthening effect of acicular ferrite and the deformation capacity generally associated with carbon-enriched austenite in ADI [10,15,18].

Tribological testing confirmed the beneficial effect of the heat treatment. Although the mean friction coefficients were similar, the maximum wear-track depth decreased from 43.5 to 3 μm and the cross-sectional area decreased from 2.98 × 10^4^ to 210 μm^2^. Thus, the improvement resulted primarily from increased resistance to penetration and material removal rather than from a reduction in friction [4,9,14,15,16,17].

Interpretations involving retained-austenite stability or strain-induced martensitic transformation remain literature-supported hypotheses because no XRD or EBSD measurements were performed. The optical observations and mechanical response are compatible with these mechanisms, but they do not constitute direct phase evidence [9,10,16].

Overall, the results show that the selected austempering cycle both improves mechanical and tribological performance and limits wall-thickness-related variations. This is technologically important for complex castings in which local solidification conditions would otherwise produce non-uniform properties.

## 4. Conclusions

1. Wall thickness strongly affected the as-cast microstructure: graphite nodule density decreased from 326 to 141 mm^−2^, while the ferrite fraction increased from 64.8% to 95.0% between the 8 and 48 mm sections.
2. Austenitization at 880–890 °C for 2.5 h followed by austempering at 370–380 °C for 2 h produced an optically uniform acicular matrix consistent with ausferrite throughout all investigated sections.
3. The heat treatment increased the average hardness to 296.4 HBW, the ultimate tensile strength to 935 MPa, and elongation to 8.6%, while narrowing the wall-thickness-related hardness variation.
4. The mean Charpy impact energy increased from 5.12 to 10.06 J, demonstrating improved impact-energy absorption after austempering.
5. Wear resistance improved markedly: the wear-track cross-sectional area decreased from approximately 2.98 × 10^4^ to 210 μm^2^ and the maximum depth decreased from 43.5 to 3 μm.
6. The selected austempering cycle provides a useful route for obtaining a more uniform combination of strength, impact resistance, and dry-sliding wear resistance in EN-GJS-800-8 castings with variable wall thickness.

## Figures and Tables

**Table 1:** Chemical composition of the investigated EN-GJS-800-8 ductile iron (wt.%).

C	Si	Mn	S	Ni	Cu	P	Mo	Mg
3.477	2.097	0.146	0.0096	2.648	0.676	0.022	0.005	0.0441

**Table 2:** Experimental matrix and parameters of the tested EN-GJS-800-8 ductile iron.

Process Parameter/Test	State: As-Cast	State: Austempered (ADI)	Applied Standard/Device
Chemical analysis	Spectrolab M10 OES	—	ASTM E1999 [22]
Austenitizing treatment	—	880–890 °C/2.5 h	Experimental laboratory cycle
Austempering	—	370–380 °C/2.0 h	Liquid salt bath cycle
Metallographic equipment	Nikon Eclipse MA100	Nikon Eclipse MA100	ASTM E3 [23]
Hardness evaluation	$\bar{x}$ = 201.4 HBW	$\bar{x}$ = 296.4 HBW	Innovatest Nexus 3300/ISO 6506-1 [19]
Tensile strength (R_m_)	567 MPa	935 MPa	Zwick/Roell Z250/ISO 6892-1 [20]
Elongation (A_5_)	6.2%	8.6%	Zwick/Roell Z250/ISO 6892-1 [20]
Charpy impact energy	4.60 J; 5.60 J	10.01 J; 10.11 J	HOYTOM HYTT 300J/ISO 148-1 [21]

**Table 6:** Charpy V-notch impact energy results for EN-GJS-800-8 ductile iron in the as-cast and austempered conditions.

Material Condition	Specimen No.	Absorbed Impact Energy, KV [J]	Mean Impact Energy [J]	Standard Deviation [J]
As-cast condition	1	4.64	5.12	0.68
	2	5.60		0.68
Austempered condition (ADI)	1	10.11	10.06	0.07
	2	10.01		0.07

**Table 7:** Stereometric parameters of wear tracks obtained for EN-GJS-800-8 ductile iron in the as-cast and austempered conditions.

Stereometric Parameter	As-Cast Condition	Austempered Condition (ADI)	Change and Interpretation
**Maximum depth [µm]**	43.5	3	Decrease by over 93%.
**Wear-track cross-sectional area [µm^2^]**	2.98 × 10^4^	210	Over 140-fold reduction in material loss.
**Maximum height [µm]**	2.8	4.1	Increase in the ADI state (microplasticity).
**Pile-up area [µm^2^]**	71.4	673.8	Strong local hardening of the trace edge.

## Data Availability

The data presented in this study are available from the corresponding author upon reasonable request.
